# Dissection and Design of Yeast Prions

**DOI:** 10.1371/journal.pbio.0020086

**Published:** 2004-03-23

**Authors:** Lev Z Osherovich, Brian S Cox, Mick F Tuite, Jonathan S Weissman

**Affiliations:** **1**Department of Cellular and Molecular Pharmacology and Howard Hughes Medical Institute, University of CaliforniaSan Francisco, CaliforniaUnited States of America; **2**Department of Biosciences, University of KentCanterburyUnited Kingdom

## Abstract

Many proteins can misfold into β-sheet-rich, self-seeding polymers (amyloids). Prions are exceptional among such aggregates in that they are also infectious. In fungi, prions are not pathogenic but rather act as epigenetic regulators of cell physiology, providing a powerful model for studying the mechanism of prion replication. We used prion-forming domains from two budding yeast proteins (Sup35p and New1p) to examine the requirements for prion formation and inheritance. In both proteins, a glutamine/asparagine-rich (Q/N-rich) tract mediates sequence-specific aggregation, while an adjacent motif, the oligopeptide repeat, is required for the replication and stable inheritance of these aggregates. Our findings help to explain why although Q/N-rich proteins are relatively common, few form heritable aggregates: prion inheritance requires both an aggregation sequence responsible for self-seeded growth and an element that permits chaperone-dependent replication of the aggregate. Using this knowledge, we have designed novel artificial prions by fusing the replication element of Sup35p to aggregation-prone sequences from other proteins, including pathogenically expanded polyglutamine.

## Abstract 2

Artificial prions - infectious, misfolded proteins - can be created by fusing the replication element of one prion to aggregation sequences from another

## Introduction

The aggregation of misfolded proteins underlies a diverse range of human diseases, including sporadic amyloidoses such as Alzheimer's disease and hereditary neuropathies such as Huntington's disease (Dobson 1999). Prions are a special class of protein aggregates that replicate their conformation and spread infectiously (Prusiner 1998). After the discovery that prion aggregates are responsible for the mammalian transmissible spongiform encephalopathies, several epigenetically heritable traits in fungi were also found to depend on a prion mechanism (Wickner 1994; Uptain and Lindquist 2002; Osherovich and Weissman 2004). In Saccharomyces cerevisiae and *Podospora anserina,* prions are transmitted from cell to cell through mating and cell division, resulting in readily assayed phenotypes with a non-Mendelian pattern of inheritance (Liebman and Derkatch 1999).

The yeast non-Mendelian factors [*PSI^+^*] (Cox 1965) and [*URE3*] (Lacroute 1971), which are prion forms of the translation termination factor Sup35p and the transcriptional activator Ure2p, respectively, have served as useful models for the formation and replication of heritable protein aggregates. Prion forms of Sup35p and Ure2p lead to defects in their respective biochemical activities (translation termination and nitrogen catabolite repression). Mutational analysis has shown the glutamine/asparagine-rich (Q/N-rich) amino-terminal (N) domains of these proteins to be critical for prion behavior (Ter-Avanesyan et al. 1993; Masison and Wickner 1995; Patino et al. 1996; Paushkin et al. 1996; DePace et al. 1998). In vitro, these Q/N-rich domains form self-seeding, β-sheet-rich amyloid fibrils similar to those associated with Alzheimer's and Huntington's diseases (Glover et al. 1997; King et al. 1997; Taylor et al. 1999). The autocatalytic aggregation of yeast prion proteins often shows a high specificity for like molecules; for example, Sup35p N domains from different yeast species form prion aggregates that preferentially interact with molecules of their own kind (Santoso et al. 2000; Chernoff et al. 2000; Kushnirov et al. 2000; Zadorskii et al. 2000; Nakayashiki et al. 2001). [*PSI^+^*] and [*URE3*] can be eliminated by transient growth in the presence of guanidine hydrochloride (GuHCl), which “cures” cells of prions by inhibiting Hsp104p, a molecular chaperone needed for prion replication (Chernoff et al. 1995; Jung et al. 2002; Ness et al. 2002).

A surprisingly large number of proteins in S. cerevisiae and other eukaryotes have lengthy Q/N-rich tracts ostensibly similar to those found in the prion-forming domains of Sup35p and Ure2p (Michelitsch and Weissman 2000). From among these, we and another group identified two novel proteins, New1p and Rnq1p, with prion-forming domains resembling those of Sup35p and Ure2p (Santoso et al. 2000; Sondheimer and Lindquist 2000). When these Q/N-rich domains were fused to green fluorescent protein (GFP) and overexpressed, they formed visible aggregates resembling those of GFP-labeled Sup35p in [*PSI^+^*] cells. Fusion proteins in which these domains were introduced in place of the Sup35p prion domain could support distinct, self-specific prion states that recapitulated the translation termination defect associated with [*PSI^+^*]. Rnq1p was later shown to underlie a naturally occurring prion called [*PIN^+^*], which promotes the aggregation of Q/N-rich proteins such as Sup35p; overexpressed Sup35p forms aggregates and stimulates the appearance of [*PSI^+^*] only in [*PIN^+^*] strains (Derkatch et al. 1997; Derkatch et al. 2001). Aggregates of the New1p prion domain, whether resulting from overexpression or from a constitutive prion form (termed [*NU^+^*]), also promoted the aggregation of other Q/N-rich proteins, emulating the effect of [*PIN^+^*] (Osherovich and Weissman 2001).

Many sequences with Q/N content as high as that of Sup35p and Ure2p, including human polyglutamine expansion disease proteins, form visible aggregates when overexpressed in yeast as GFP fusions (Krobitsch and Lindquist 2000; Osherovich and Weissman 2001; Meriin et al. 2002). However, only a limited number of Q/N-rich sequences are bone fide prion domains capable of propagating these aggregates over multiple cell generations even when expressed at low levels (J. Hood and J.S.W, unpublished data). To understand what distinguishes generic Q/N-rich aggregates from heritable prions, we conducted a detailed dissection of the prion-forming regions of Sup35p and New1p. We found that the prion properties of Sup35p and New1p require the presence of two independent and portable sequence elements within their prion domains. One element mediates the growth of prion aggregates by incorporation of soluble monomers. The second promotes the inheritance of aggregates, generating new heritable “seeds” which can be partitioned between mother and daughter cells during cell division.

## Results

### Distinct Regions of the New1p Prion Domain Mediate Prion Growth and Division

**Figure 1:**
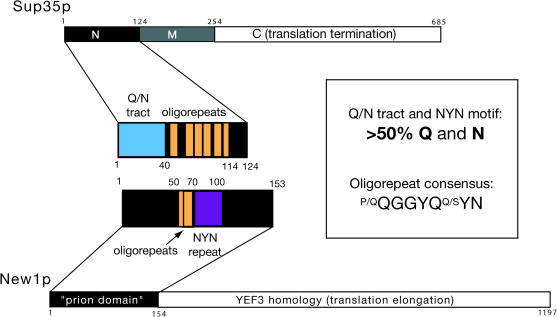
Schematic Diagram of Sup35p and New1p Prion domains of both proteins are enlarged in the center, highlighting the Q/N-rich tract of Sup35p (blue), the NYN tripeptide repeat of New1p (purple), and the oligopeptide repeat sequences (orange) found in both proteins. The sequence of the NEW1 oligopetide repeat (residues 50–70) is QQQRNWKQGGNYQQGGYQSYN, while that of the adjacent tripeptide repeat region (residues 71–100) is SNYNNYNNYNNYNNYNNYNNYNKYNGQGYQ.

Sup35p can alternate between a biochemically active, soluble form ([*psi^–^*]) and an aggregated prion state ([*PSI^+^*]) with diminished translation termination activity, which can be monitored by nonsense suppression of the mutant *ade1–14* allele (Liebman and Derkatch 1999). Whereas [*psi^–^*] strains form red colonies on yeast extract-peptone-dextrose (YEPD) medium and cannot grow in the absence of adenine, [*PSI^+^*] strains suppress the premature stop codon in *ade1-14*, and thus appear pink or white on YEPD medium and grow on adenine-free medium (a phenotype termed adenine prototrophy, Ade+). The N or prion domain of Sup35p (residues 1-112) is required for [*PSI^+^*] formation but is dispensable for the translation termination activity of the carboxy-terminal C domain (Ter-Avanesyan et al. 1993). The charged middle domain (M) is not required for prion behavior, but modulates the efficiency of chaperone-dependent prion transmission (Liu et al. 2002; L.Z.O., unpublished data) (Figure 1). Two distinct regions in the N domain have previously been implicated in Sup35p aggregation: a Q/N-rich tract (residues 1–39) (DePace et al. 1998) and an oligopeptide repeat (residues 40–112) that consists of five and a half degenerate repeats of the consensus sequence P/QQGGYQQ/SYN (Liu and Lindquist 1999; Parham et al. 2001; Crist et al. 2003).

We had earlier identified New1p as an uncharacterized protein with a Sup35p-like N-terminal domain; when fused to the M and C domains of Sup35p, the first 153 residues of New1p (New1_1–153_) supported a [*PSI^+^*]-like prion state termed [*NU^+^*] (Santoso et al. 2000). Sup35p and New1p have regions of clear similarity beyond their high Q/N content (Figure 1). The prion domains of both have Q/N-rich tracts and oligopeptide repeat regions, although their order is reversed. The C-terminal domains of New1p and Sup35p also appear to be related, based on modest homology and the similarity of the translation termination defects in *sup35* (Song and Liebman 1985) and *new1* mutants (L.Z.O., unpublished data).

**Figure 2:**
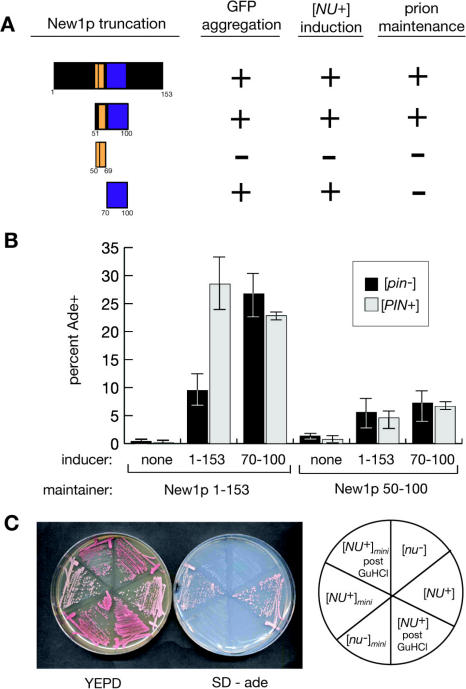
Dissection of the New1p Prion Domain Reveals Distinct Regions Responsible for Aggregation and Prion Inheritance (A) Indicated fragments of New1p (left) were expressed as GFP fusions (inducers) in a [*nu^–^*] [*pin^–^*] strain, examined by microscopy for GFP aggregation, then plated on SD-ade medium to assess induction of [*NU^+^*]. The symbol “+” indicates induction frequencies of at least 5%; the symbol “–” indicates no induction. Maintenance was assessed by the ability of an episomal maintainer version of the indicated fragment to support an Ade+ state after overexpression of New1_1–153_-GFP (see Materials and Methods). The aggregation of New1-GFP fusions (second column) has been previously reported (Osherovich and Weissman 2001). (B) The NYN repeat of New1p induces [*NU^+^*] and [*NU^+^*]*_mini_.* New1_70–100_-GFP was overexpressed in [*nu^–^*] and [*nu^–^*]*_mini_* strains ([*pin^–^*] and [*PIN^+^*] derivatives of each), along with vector only or New1_1–153_-GFP controls. Averages of three independent trials, representing 600–2000 colonies, are shown for most induction experiments; inductions using New1_70–100_-GFP were conducted twice. Error bars show minimal and maximal observed induction efficiencies. (C) Reversibility of [*NU^+^*]*_mini_.* The [*pin^–^*] Ade+ convertants obtained in (B) were colony purified on SD-ade medium and confirmed to have lost the inducer plasmid. A stable [*NU^+^*]*_mini_* isolate is shown before and after induction, as well as after GuHCl treatment, along with [*nu^–^*] and [*NU^+^*] reference strains.

To understand the sequence requirements for the prion behavior of New1p, we constructed a series of truncated prion domains (Figure 2A) and examined their participation in several critical steps of the prion replication cycle. We first asked whether these truncated prion domains could form visible foci when fused to GFP (aggregation). Next, we examined whether such aggregates could convert New1_1–153_ into a [*NU^+^*] prion state (induction). Finally, we fused these constructs to the M and C domains of Sup35p (–M-C), introduced them in place of endogenous SUP35, and assessed whether these proteins could adopt stable prion states (maintenance).

We found that distinct regions within the New1p prion domain are necessary for the induction and maintenance of [*NU^+^*] (Figure 2A). The asparagine-tyrosine-asparagine (NYN) repeat (residues 70–100), which we had earlier shown to be sufficient for aggregation (Osherovich and Weissman 2001), also proved sufficient for induction of [*NU^+^*]. As with the full-length New1p prion domain, overexpression of the NYN repeat efficiently stimulated the appearance of Ade+ in [*nu^–^*] cells (Figure 2B, left). However, stable prion maintenance required both the NYN repeat and the adjacent oligopeptide repeat. In a strain with this minimized New1p prion domain (residues 50–100), overexpression of the full prion domain or of the NYN repeat alone promoted the appearance of Ade+ colonies (Figure 2B, right). The resulting convertants remained Ade+ after loss of the inducer plasmid but reverted to Ade- after transient GuHCl treatment (Figure 2C). We conclude that the oligopeptide repeat and the NYN repeat of New1p together are sufficient to support a prion state, termed [*NU^+^*]*_mini_,* which recapitulates the characteristics of [*NU^+^*].

### Dissection of the Sup35p Prion Domain

**Figure 3:**
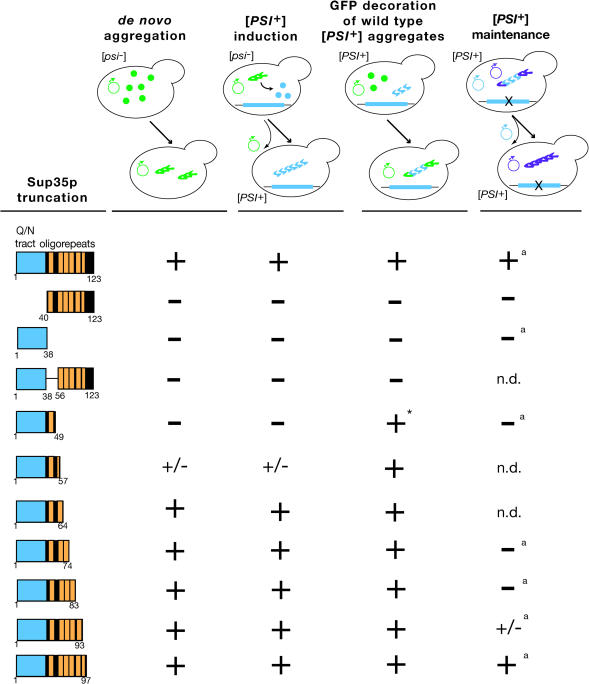
Dissection of the Sup35p Prion Domain At top are schematic diagrams of these experiments; positive outcomes are shown below the arrows. In some cases, similar experiments have been reported by Parham et al. (2001) (indicated by “a”) and are repeated here as controls. Aggregation: Plasmid-borne M-GFP fusions of the indicated Sup35p N domain fragments (green) were overexpressed in a [*psi^–^*] [*PIN^+^*] strain and examined for fluorescent focus formation. The symbol “+” indicates that 10% or more of cells displayed aggregates. Sup35_1–57_-M-GFP displayed a lower frequency of aggregation (approximately 1%). Induction: Strains from the aggregation experiment were plated onto SD-ade medium and scored for growth to test whether aggregates of truncated protein (green) convert chromosomally encoded protein (blue) to [*PSI^+^*]. The symbol “+” indicates approximately 5–10% conversion frequency. Consistent with the aggregation experiment, Sup35_1–57_-M-GFP displayed a lower frequency of [*PSI^+^*] induction (approximately 1%). Decoration: Indicated proteins were expressed as –M-GFP fusions in [*PSI^+^*] [*PIN^+^*] cells, which were examined to determine whether GFP-labeled truncations (green) decorate preexisting aggregates of full-length Sup35p (blue). Curiously, Sup35_1–49_-M-GFP in [*PSI^+^*] cells formed abnormally large “ribbon” aggregates of the kind typically observed during de novo [*PSI^+^*] induction; furthermore, approximately 10% of the cells reverted to [*psi^–^*] (indicated by “*”). Thus, this truncation was a potent dominant PNM mutant. Maintenance: A SUP35-deleted [*PSI^+^*] [*PIN^+^*] bearing wild-type SUP35 maintainer (blue) was transformed with maintainer plasmids containing the indicated truncation (purple). The wild-type maintainer was lost by counterselection, and the resulting strain was tested for [*PSI^+^*] by color and growth on SD-ade medium. The Sup35_1–93_ mutant displayed an intermediate pink color and grew poorly on SD-ade medium, as previously reported (Parham et al. 2001). Note: King (2001) reports that Sup35_1–61_-GFP fusion could decorate [*PSI^+^*] aggregates in certain strains and could induce [*PSI^+^*] de novo when overexpressed.

In light of the similarity between New1p and Sup35p prion domains, we asked whether separate regions of Sup35p were involved in the induction and maintenance of [*PSI^+^*] aggregates (Figure 3). We constructed a series of truncated Sup35p N domains and analyzed their behavior in the aggregation, induction, and maintenance assays described above for [*NU^+^*]. Additionally, we examined the ability of truncated N domains to decorate preexisting Sup35p aggregates in [*PSI^+^*] strains.

We found that the Q/N-rich tract and a small portion of the adjacent oligopeptide repeat are responsible for Sup35p aggregation and de novo [*PSI^+^*] induction. Deletions within the Q/N-rich tract or of oligopeptide repeat 1 abolished these properties, whereas a construct containing only the Q/N-rich region and the first two oligopeptide repeats (residues 1–64) aggregated and induced [*PSI^+^*] at levels comparable to the full prion domain, in agreement with King (2001). A construct (residues 1–57) with a partial deletion of oligopeptide repeat 2 could still aggregate and induce [*PSI^+^*], albeit with decreased efficiency. Although a construct lacking oligopeptide repeat 2 entirely (residues 1–49) did not induce [*PSI^+^*] de novo, this GFP fusion could nonetheless decorate preexisting Sup35p aggregates. Thus, while oligopeptide repeat 2 contributes to the aggregation of Sup35p, the primary determinants of prion induction reside in the amino-terminal Q/N-rich region and oligopeptide repeat 1.

In contrast, the rest of the oligopeptide repeat region is needed for stable inheritance of [*PSI^+^*] aggregates. Constructs that did not form fluorescent foci could not retain [*PSI^+^*], suggesting that aggregation is a prerequisite for prion maintenance. However, aggregation is not sufficient for prion inheritance, as Sup35p constructs with deletions spanning oligopeptide repeats 3–5 could not support a prion state despite their ability to form aggregates and efficiently induce [*PSI^+^*]. Only the sixth (incomplete) oligopeptide repeat proved dispensable for [*PSI^+^*] maintenance, consistent with an earlier report (Parham et al. 2001).

### The PNM2-1 Mutation in Oligopeptide Repeat 2 Specifically Compromises the Inheritance of [*PSI^+^*]

Our deletion analysis suggested that oligopeptide repeat 2 participated in both the formation and inheritance of Sup35p aggregates. We made use of a point mutation within oligopeptide repeat 2 known as PNM2-1 (G58D) to distinguish between these two functions. PNM2-1 (**P**SI **N**o **M**ore) shows strong interference with [*PSI^+^*] in certain strain backgrounds through a poorly understood mechanism (McCready et al. 1977; Doel et al. 1994; Kochneva-Pervukhova et al. 1998; Derkatch et al. 1999).

**Figure 4:**
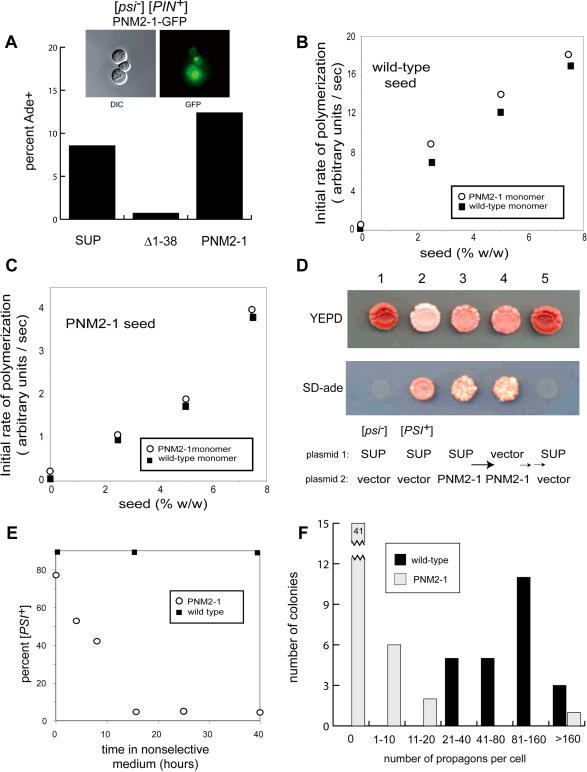
PNM2–1 (G58D) Prevents Inheritance But Not Aggregation of Sup35p Prions (A) PNM2-1 protein can seed [*PSI^+^*]. A Sup35p inducer containing the PNM2-1 (G58D) mutation was overexpressed in [*psi^–^*] [*PIN^+^*] cells; shown are cells (inset) with representative fluorescent foci, which were the same in frequency and appearance as cells with a wild-type inducer. Cells overexpressing inducer versions of wild-type Sup35p (SUP), an aggregation-defective N-terminal truncation (Δ1–38), and PNM2-1 were plated and scored for Ade+. Approximately 1000 colonies were counted. (B) PNM2-1 protein polymerization is similar to that of wild-type protein. (C) Preformed PNM2-1 polymers seed wild-type and PNM2-1 monomers with comparable efficiency. Endpoint PNM2-1 polymers were used to seed fresh reactions. (D) PNM2-1 displays a partially dominant, incompletely penetrant defect in [*PSI^+^*] maintenance. [*psi^–^*] (1) and [*PSI^+^*] (2) SUP35::TRP1 pSUP35 controls are shown. [*PSI^+^*] [*PIN^+^*] SUP35::TRP1 pSUP35 was transformed with a second maintainer expressing PNM2-1 (3). The wild-type maintainer (pSUP35) was then lost through counterselection (4). Red sectors from (4) were isolated, retransformed with the wild-type maintainer, and allowed to lose the PNM2-1 maintainer (5). (E) Mitotic instability of [*PSI^+^*] in the PNM2-1 strain. A pink (Ade+) [*PSI^+^*] [*PIN^+^*] PNM2-1 isolate was grown to log phase in SD-ade liquid then shifted into nonselective (YEPD) medium. At indicated time points, aliquots were plated onto SD-ade and YEPD media to determine the fraction of [*PSI^+^*] cells (minimum of 200 colonies counted per time point). Whereas a wild-type control remained [*PSI^+^*] through the experiment, the PNM2-1 strain rapidly lost [*PSI^+^*] during logarithmic growth; during stationary phase (18 h and beyond), the percentage of [*PSI^+^*] PNM2-1 strains remained unchanged (approximately 5%). (F) Propagon count of PNM2-1 vs. wild-type [*PSI^+^*] strains. The majority of PNM2-1 cells had no [*PSI^+^*] propagons (i.e., were [*psi^–^*]). In both strains, a small number of “jackpot” cells contained over 200 propagons; see Cox et al. (2003).

Using both in vivo and in vitro assays, we established that PNM2-1 does not have a defect in aggregation or [*PSI^+^*] induction. Earlier work indicated that PNM2-1 is capable of seeding [*PSI^+^*] in vivo (Kochneva-Pervukhova et al. 1998; Derkatch et al. 1999; King 2001). Consistent with these reports, we found that overexpression of a PNM2-1-GFP fusion in [*psi^–^*] [*PIN^+^*] cells with a wild-type *SUP35* locus led to both focus formation and [*PSI^+^*] induction (Figure 4A). A previous study of Sup35p polymerization in extracts had suggested that PNM2-1 might interfere with [*PSI^+^*] through a defect in seeding (Kochneva-Pervukhova et al. 1998). We tested this by examining the rate of seeded polymerization of recombinant PNM2-1 protein. Like wild-type Sup35p, purified PNM2-1 spontaneously formed amyloid fibrils in vitro; this was accelerated by the addition of preformed Sup35p polymer seeds (data not shown). We measured the initial rates of polymerization of wild-type and PNM2-1 protein seeded by preformed wild-type polymers (Figure 4B) and by PNM2-1 polymers (Figure 4C) using a thioflavin-T–binding assay. We observed that wild-type and PNM2-1 monomers were seeded by wild-type polymers with similar kinetics; likewise, PNM2-1 polymers seeded both wild-type and PNM2-1 monomers equivalently. Thus, the PNM2-1 mutation does not affect polymerization or seeding.

Instead, the PNM2-1 strain shows a marked defect in the inheritance of [*PSI^+^*]. When the wild-type *SUP35* gene of a [*PSI^+^*] strain was replaced with PNM2-1, the strain retained the prion on synthetic defined (SD) yeast medium that selected for [*PSI^+^*] (SD-ade medium) but reverted to [*psi^–^*] at a high frequency in nonselective YEPD medium, resulting in sectored colonies (Figure 4D). We measured the rate of [*PSI^+^*] loss in a PNM2-1 strain by growing it in YEPD medium and, at various time points, plating aliquots of the culture onto SD-ade medium to determine the fraction of cells that had retained [*PSI^+^*] (Figure 4E). A wild-type strain retained [*PSI^+^*] in all of the cells throughout the experiment. By contrast, in the PNM2-1 strain the fraction of [*PSI^+^*] cells decreased rapidly while the cells grew logarithmically, but remained at a constant level when the cells entered stationary phase. These findings indicate that PNM2-1 acts to eliminate [*PSI^+^*] in dividing cells, consistent with a defect in prion replication.

We next used a recently described assay to measure the number of heritable prion seeds (propagons) in a PNM2-1 strain. Here, prion replication is inhibited by GuHCl treatment. As the cells divide, preexisting propagons are diluted but not destroyed. The number of propagons present in a colony arising from a single cell is then evaluated by removing the GuHCl prion replication block after a large number (10 or more) of cell divisions and counting the total number of [*PSI^+^*] cells in that colony (Cox et al. 2003). Whereas a wild-type strain had a median of 92 (*n* = 24) propagons per cell, the PNM2-1 strain had dramatically fewer: 41 of 50 cells had no [*PSI^+^*] propagons at all (i.e., were [*psi^–^*]), and among the remaining nine [*PSI^+^*] cells, the median propagon number was six (Figure 4F). Thus, although a PNM2-1 strain can harbor [*PSI^+^*] prions, a defect in propagon replication causes mitotic instability, demonstrating the importance of oligopeptide repeat 2 in prion replication or segregation.

### Design of Novel Prion Domains

**Figure 5:**
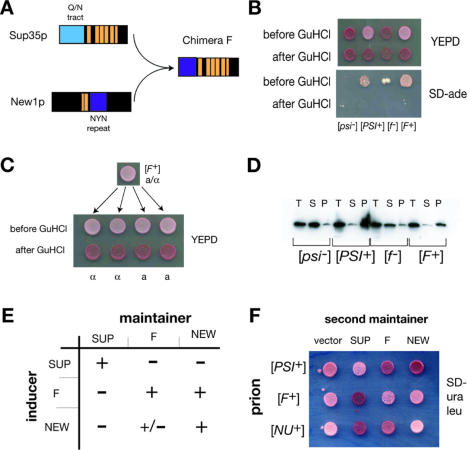
F, A New1p–Sup35p Chimera, Shows Prion Characteristics of New1p (A) Schematic diagram illustrating the construction of chimera F. (B) Chimera F forms a prion, [*F^+^*]. The *SUP35* gene in a [*psi^–^*] [*pin^–^*] strain was replaced with the F-M-C fusion; after transient overexpression of F-M-GFP, approximately 10% of these cells converted from an Ade- ([*f ^–^*]) to an Ade+ ([*F^+^*]) state. Shown are examples of[*f ^–^*] and [*F^+^*] strains, before and after GuHCl treatment, along with [*psi^–^*] and [*PSI^+^*] controls. (C) Non-Mendelian inheritance of [*F^+^*]. A diploid made by mating a [*F^+^*] MAT**a** strain against an [*f ^–^*] MAT**α** displayed a [*F^+^*] phenotype and, when sporulated, produced four [*F^+^*] meiotic progeny. All 11 tetrads examined showed this 4:0 pattern of inheritance. (D) Sedimentation analysis of F-M-C. Extracts of [*f ^–^*] and [*F^+^*] strains, along with [*psi^–^*] and [*PSI^+^*] controls, were subjected to 50K × g ultracentrifugation for 15 min. Total, supernatant, and pellet fractions were separated by SDS-PAGE, transferred to nitrocellulose, and probed with anti-SUP35NM serum. As with Sup35p, the prion form of F-M-C sediments primarily to the pellet but remains in the supernatant in [*f ^–^*]. (E) F-M-GFP overexpression induces [*NU^+^*] but not [*PSI^+^*]. Indicated inducers and maintainers were used in an induction experiment. The symbol “+” indicates approximately 5–10% conversion to Ade+. F induced [*NU^+^*] at a comparable efficiency to New1_1–153_; although New1_1–153_ overexpression promoted the appearance of Ade+ colonies in the F-M-C strain, these were fewer in number (less than 5%) and reverted to Ade- after restreaking. (F) [*F^+^*] and [*NU^+^*] prion proteins interact with each other but not with [*PSI^+^*]. Episomal “second maintainers” were introduced into the indicated strains, along with an empty vector control. Antisuppression (red) indicates that the second maintainer is soluble, while white/pink indicates coaggregation of the endogenous and episomal maintainers.

Our data suggested that the formation and inheritance of prions involve distinct regions of Sup35p and New1p prion domains. To assess the interchangeability of these prion domain components, we constructed a chimeric prion domain, termed F, in which the aggregation-determining NYN repeat of New1p was fused to the oligopeptide repeats of Sup35p (Figure 5A). While initially soluble and active, a fusion of F and the Sup35p M and C domains (F-M-C) could be converted into an aggregated state, termed [*F^+^*], after transient overexpression of F-M-GFP. As with [*NU^+^*], [*F^+^*] induction did not require [*PIN^+^*] (data not shown). [*F^+^*] could be eliminated by GuHCl treatment (Figure 5B) and was inherited in a dominant, non-Mendelian manner (Figure 5C). As with Sup35p in a [*PSI^+^*] strain, F-M-C protein in [*F^+^*] but not in [*f ^–^*] extracts sedimented entirely to the pellet fraction following high-speed centrifugation (Figure 5D). Thus, [*F^+^*] results from a prion state of F-M-C.

We next explored the specificity of [*F^+^*] prion seeding. Overexpression of the Sup35p prion domain did not induce [*F^+^*]; conversely, F-M-GFP overexpression did not induce [*PSI^+^*] (Figure 5E). However, F-M-GFP readily induced [*NU^+^*], indicating that mismatched sequences outside of the aggregating region did not prevent cross-interactions between heterologous proteins. Interestingly, overexpression of New1_1–53_-GFP induced Ade+ colonies in the [*f ^–^*] strain, but this adenine prototrophy proved unstable. We also examined the ability of preexisting prion aggregates to recruit different prion-forming proteins using an antisuppression assay (Santoso et al. 2000) (Figure 5F). [*PSI^+^*], [*F^+^*], and [*NU^+^*] strains were transformed with Sup35p–, F-M-C– or New1_1–153_-M-C–encoding plasmids; the color of the resulting colonies indicates whether the second maintainer protein is soluble (red) or aggregates as a result of the resident prion (pink/white). Consistent with the induction data, F-M-C and New1_1–153_-M-C were not incorporated into [*PSI^+^*] aggregates; likewise, Sup35p did not interact with [*F^+^*] or [*NU^+^*] aggregates. However, [*F^+^*] prions recruited New1_1–153_-M-C and, to a lesser extent, [*NU^+^*] recruited F-M-C. Thus, F and New1p prion domains can cross-interact during de novo induction and at normal levels of expression, indicating that the NYN repeat is sufficient to specify homotypic interaction between two otherwise distinct prion domains.

**Figure 6:**
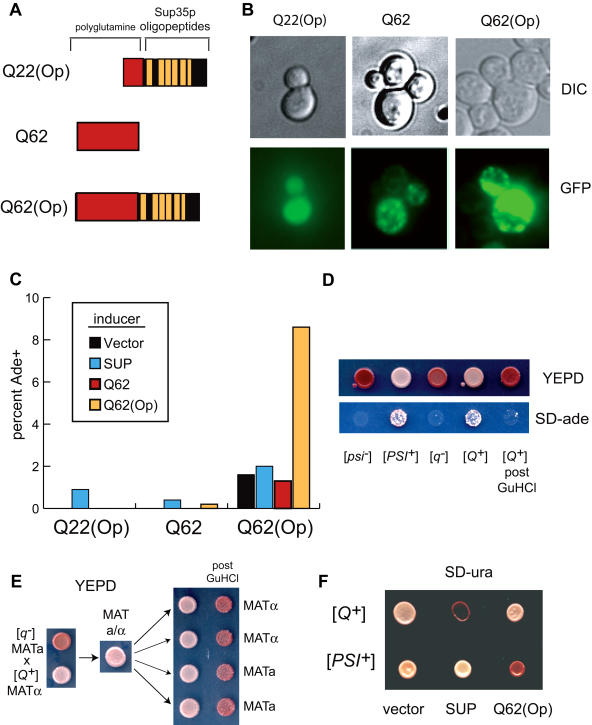
[*Q^+^*], a Prion Form of Pathogenically Expanded Polyglutamine (A) Schematic illustrating the construction of polyglutamine-derived prion domains. (Op) indicates the presence of the Sup35p oligopeptide repeats (residues 40–124). (B) Fluorescence micrographs of [*psi^–^*] [*PIN^+^*] strains expressing indicated polyglutamine inducers. (C) Polyglutamine-based prion inheritance. Strains with indicated inducers and maintainers were plated onto SD-ade and YEPD media to determine the fraction of Ade+ after a standard induction experiment. Interestingly, Q62 inducer forms aggregates but does not promote Ade+ in the Q62(Op) maintainer strain. Note that Q62(Op) shows a high rate of spontaneous appearance of Ade+. (D) GuHCl sensitivity of the [*Q^+^*] state. An Ade+ convertant obtained in (C) was restreaked to lose the inducer plasmid, then grown on GuHCl. Shown are plates before and after GuHCl treatment, along with [*psi^–^*] and [*PSI^+^*] controls. (E) Dominance and non-Mendelian inheritance of [*Q^+^*]. See Figure 5C. (F) [*Q^+^*] does not interact with Sup35p and vice versa. [*Q^+^*] and [*PSI^+^*] strains were transformed with indicated maintainers; mismatches between the maintainer and the chromosomally encoded allele result in antisuppression (red).

Can a simple aggregation-prone sequence such as polyglu-tamine (Zoghbi and Orr 2000) be turned into a heritable prion by fusion to an oligopeptide repeat? We designed artificial prion domains containing short (Q22) and pathogenically expanded (Q62) polyglutamine tracts, either alone or adjacent to the Sup35p oligopeptide repeat (Figure 6A). These domains were fused to –M-GFP and –M-C to create polyglutamine inducers and maintainers, respectively. Q22 inducers did not aggregate upon overexpression, but Q62 inducers (with and without oligopeptide repeats) formed visible foci in [*psi^–^*] [*PIN^+^*] cells (Figure 6B). Transient overexpression of Q62 inducers had no effect on the Q22 maintainer with the oligopeptide repeat or on the Q62 maintainer lacking the oligopeptide repeat. However, the Q62 maintainer with an oligopeptide repeat supported prion inheritance, converting to a stable Ade+ state following overexpression of the cognate inducer (Figure 6C). Several tests confirmed the prion nature of this state, termed [*Q^+^*]. Like [*PSI^+^*], [*Q^+^*] did not require the presence of the inducer plasmid after transient overexpression, was sensitive to GuHCl treatment (Figure 6D), and displayed a dominant, non-Mendelian pattern of inheritance (Figure 6E). We further tested the specificity of the [*Q^+^*] state by introducing a plasmid encoding a noncognate second maintainer into a [*Q^+^*] strain (Figure 6F). The Q62 maintainer failed to be incorporated into [*PSI^+^*] aggregates, causing antisuppression (red); conversely, Sup35p did not enter [*Q^+^*] aggregates.

## Discussion

A number of epigenetic traits in fungi result from the stable inheritance of self-propagating, infectious protein aggregrates (prions) (Uptain and Lindquist 2002). Prion inheritance requires three sequential events that must keep pace with cell division to preserve the number of heritable prion units, or propagons, per cell (Osherovich and Weissman 2004). First, prion aggregates must grow in size by incorporating newly synthesized protein. Next, these enlarged aggregates must be divided into smaller ones through the action of cellular chaperones (Kushnirov and Ter-Avanesyan 1998; Borchsenius et al. 2001; Ness et al. 2002; Kryndushkin et al. 2003). Finally, these regenerated propagons must be distributed to mother and daughter cells (Cox et al. 2003); for small, cytoplasmic aggregates, this distribution may occur passively by diffusion during cytokinesis. In the present study, we have dissected the prion-forming domains of Sup35p and New1p to discover the sequence elements involved in these steps. We have found that these domains consist largely of modular, interchangeable elements that serve distinct functions of prion growth and division or transmission.

**Figure 7:**
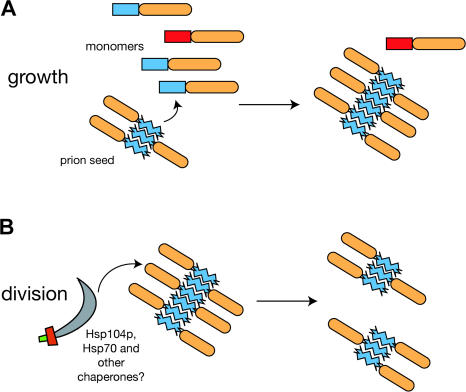
Model for Prion Growth and Division (A) During prion growth, polymers seed the incorporation of monomers through interactions between Q/N-rich aggregation sequences (blue). Proteins with noncognate aggregation sequences (red) are excluded. (B) The division phase of prion replication requires the oligopeptide repeats (orange), which may facilitate the action of chaperones such as Hsp104p (scimitar) in breaking the polymer into smaller, heritable units.

Aggregation underlies the growth phase of the prion replication cycle (Figure 7A) and occurs through the templated addition of conformationally compatible monomers onto preexisting seeds. Like other amyloids, yeast prions display a high specificity for homotypic aggregation (Santoso et al. 2000; Chernoff et al. 2000; Kushnirov et al. 2000; Zadorskii et al. 2000; Nakayashiki et al. 2001). This discrimination arises from differences in the amino acid sequence and the conformation (Chien and Weissman 2001) of the aggregation-promoting Q/N-rich elements found in each yeast prion protein. Aggregation and specificity are dictated by the NYN repeat (residues 70–100) of New1p and by the Q/N-rich amino terminal region (residues 1–57) of Sup35p.

In contrast, the conserved oligopeptide repeat sequence mediates the division and/or segregation phase of prion replication (Figure 7B). In New1p, the NYN repeat alone can aggregate and induce [*NU^+^*] but requires an adjacent oligopeptide repeat to form a minimal heritable New1p prion, [*NU^+^*]*_mini_.* Similarly, in Sup35p, the Q/N-rich amino terminal region mediates aggregation whereas most of the oligopeptide repeats are needed for the inheritance of [*PSI^+^*] propagons. Oligopeptide repeats 1 and 2 appear to contribute to both growth and inheritance, consistent with earlier reports that expansion and deletion within this region modulate in vitro polymerization of Sup35p and the appearance of [*PSI^+^*] in vivo (Liu and Lindquist 1999). However, the two functions can be distinguished by a point mutant in oligopeptide repeat 2 (PNM2-1), which displays a specific defect in [*PSI^+^*] inheritance despite normal aggregation. Certain [*PSI^+^*] variants are resistant to the dominant negative effect of PNM2-1 (Derkatch et al. 1999; King 2001); this suggests that although oligopeptide repeat 2 is critical for the replication of the [*PSI^+^*] variant used in our studies, it may be less important for the replication of other Sup35p prion conformations.

Many studies have established that prion inheritance requires the action of cellular chaperones such as Hsp104p and Hsp70 proteins (reviewed in Osherovich and Weissman 2002), although how these proteins contribute is poorly understood. We propose that oligopeptide repeats turn nonheritable aggregates into prions by facilitating chaperone-mediated division. Oligopeptide repeats may allow the division of aggregates by providing direct binding sites for chaperones or by altering the conformation of the amyloid core to allow chaperone access. An earlier study established that deletion of residues 22–69 of Sup35p (which include parts of both the Q/N tract and the oligopeptide repeat) interferes with both [*PSI^+^*] induction and chaperone-mediated prion disaggregation (Borchsenius et al. 2001). Unlike the Δ22–69 mutant, the prion replication defect in PNM2-1 could not be corrected by increasing Hsp104p levels (data not shown), arguing that the mitotic instability of PNM2-1 [*PSI^+^*] is not due solely to inadequate Hsp104p binding.

Our findings help to explain why, among many Q/N-rich proteins in yeast, only a small subset form heritable prions. While many Q/N-rich proteins can aggregate when overexpressed (Sondheimer and Lindquist 2000; Derkatch et al. 2001; Osherovich and Weissman 2001), prion inheritance of such aggregates requires that they be divided and passed on to the next generation. Although the inheritance of Sup35p and New1p prions is mediated by oligopeptide repeats, other sequences could also serve this purpose. Ure2p lacks an oligopeptide repeat; interestingly, many isolates of [*URE3*] are mitotically unstable in the absence of selection (Schlumpberger et al. 2001). Rnq1p, which underlies [*PIN^+^*], also lacks a strict oligopeptide repeat, but a region (residues 218–405) within its prion domain has an amino acid content reminiscent of the oligopeptide repeat sequence (i.e., numerous Q, N, S, Y, and G residues) (Resende et al. 2003). Only two other yeast proteins, YDR210W and YBR016W, have clearly recognizable oligopeptide repeats; both proteins also have Q/N-rich regions. YBR016W forms aggregates when overexpressed (Sondheimer and Lindquist 2000), but it is not known whether either protein can maintain a heritable aggregated state. Although the mammalian prion protein PrP contains a sequence resembling the oligopeptide repeat that can functionally replace one of the Sup35p repeats (Parham et al. 2001), it is unclear whether this sequence is important in the replication of the PrP^Sc^ state.

The interchangeable nature of prion domain components allowed us to design novel artificial prions. The F chimera, consisting of the aggregation sequence of New1p and the oligopeptide repeat of Sup35p, demonstrates that the growth and specificity of prions is largely determined by the Q/N-rich tract, not by the oligopeptide repeat. Despite a sequence derived primarily from Sup35p, the F chimera behaved like New1p rather than like Sup35p. The [*F^+^*] prion cross-interacted with New1p but not Sup35p. Like [*NU^+^*], [*F^+^*] could be induced in the absence of a prion-promoting (PIN) factor. Finally, [*F^+^*] could itself act as a PIN factor, as does [*NU^+^*] (data not shown). Notably, the NYN repeat of New1p functions as an aggregation module apparently without regard to its position within a protein; this sequence induced prions when overexpressed by itself or with oligopeptide repeats at its N-terminal (in New1_1–153_ and New1_50–100_) or C-terminal regions (in the F chimera). These results suggest that aggregation sequences are portable and functionally separable from the oligopeptide repeat, perhaps constituting a structurally discrete amyloid core. Indeed, a peptide derived from the amino-terminal region of Sup35p forms a self-seeding amyloid in vitro (Balbirnie et al. 2001). A simple aggregation-prone sequence, pathogenically expanded glutamine, also supports prion inheritance when adjacent to the oligopeptide repeat, suggesting that prion domains can consist of little more than a generic, aggregating core sequence and an inheritance-promoting element.

In addition to illuminating the principles of yeast prion domain architecture, artificial prions with distinct specificity may be useful as controllable epigenetic regulators of protein activity. Such prion “switches” can be turned on and off by transient overexpression and genetic repression; for example, the Q prion domain could be fused to other proteins in order to conditionally and reversibly inactivate them independently of [*PSI^+^*]. It may also be possible to design additional artificial yeast prion domains whose aggregation is driven by non-Q/N-rich amyloidogenic proteins such as the Aβ peptide that accumulates in Alzheimer's disease (Koo et al. 1999) or the mammalian prion protein PrP (Cohen and Prusiner 1998). Such artificial prions could serve as models for aggregate–chaperone interactions in metazoans and could provide a genetic system for the high-throughput screening of modulators of human aggregation diseases.

## Materials and Methods

### 

#### Yeast strains and methods

Derivatives of W303 (Osherovich and Weissman 2001), with the initial genotypes *ade1-14, his3-11,15, leu2-3, trp1-1,* and *ura3-1,* were used throughout unless otherwise noted; all strains were [*PIN^+^*]. Strain numbers, with indicated genotypic differences, are as follows: YJW 584 [*psi^–^*] MAT**a**, YJW 508 [*PSI^+^*] MAT**α**, YJW 716 [*nu^–^*] MAT**α** *sup35*::TRP1 pRS315SpNew1_1–153_-M-C, YJW 717 [*NU^+^*] MAT**α** *sup35*::TRP1 pRS315SpNew1_1–153_-M-C, YJW 844 [*f ^–^*] MAT**α** *sup35*::F-M-C *C.g.* HIS3, YJW 881 [*F^+^*] MAT**a** *sup35*::F-M-C *C.g.* HIS3, YJW 867 [*q^–^*] MAT**α** *sup35*::Q-M-C *C.g.* HIS3, YJW 868 [*Q^+^*] MAT**a** *sup35*::Q-M-C *C.g.* HIS3. Maintainer plasmids used in Figure 3 (see plasmid and gene replacement construction, below) were introduced by plasmid shuffling into YJW 716 or YJW 753 ([*PSI^+^*] MAT**a** *sup35*::TRP1 pRS316SpSUP35), followed by loss of the maintainer spontaneously or through 5-FOA counterselection. The PNM2-1 strain in Figure 4 was generated in this manner and was subsequently restreaked on SD-ade to select for [*PSI^+^*]. HIS3-marked oligopeptide repeat truncations and PNM2-1 maintainers were from Parham et al. (2001); all other Sup35p and New1p maintainers were marked with LEU2. The [*f^–^*] strain was generated by “gamma” chromosomal integration of pRS306 F-M-C into the *SUP35* locus of YJW 584; excision of the wild-type gene was confirmed by PCR of Ade- colonies arising from subsequent growth on 5-FOA. The [*q^–^*] strain was made by “omega” chromosomal gene replacement (Kitada et al. 1995) of *SUP35* with a *C.glabrata* HIS3-marked –M-C variant (with or without oligopeptide repeats) into the *SUP35* locus of a diploid [*PSI^+^*] [*PIN^+^*] strain. After sporulation, gene replacement was confirmed by PCR and by loss of [*PSI^+^*] in half of the haploid progeny. Yeast culture methods were according to standard procedures (Sherman 1991), but YEPD-medium plates contained 1/4 of the standard amount of yeast extract to accentuate color phenotypes. For prion curing, strains were grown on YEPD medium plus 3 mM GuHCl, then restreaked onto YEPD medium.

#### Plasmid and gene replacement construction

The modular *SUP35* cloning system described in previous reports was used throughout (Santoso et al. 2000; Osherovich and Weissman 2001). All plasmids are derived from Sikorski and Hieter (1989); sequence files of all constructs are available as a web supplement (Data S1). Maintainer plasmids are low-copy CEN/ARS (pRS31x series) with the native SUP35 promoter (Sp) driving the expression of the indicated prion domain followed by the M and C domains of Sup35p. Inducer plasmids are high-copy 2μ (pRS42x series) with the inducible CUP1 promoter (Cp) driving the expression of the indicated prion domain followed by the Sup35p M domain and GFP. New1p inducers did not include the Sup35p M domain. For polyglutamine constructs, polyglutamine tracts (22 and 62) were amplified out of the MJDtr constructs used in an earlier study (Osherovich and Weissman 2001). To permit amplification, primers contained sequences homologous to several codons adjacent to the 5′ and 3′ ends of the polyglutamine tracts plus an initiator ATG codon. Thus, the polyglutamine sequences read MAYFEK(Q22/62)DLSG. The resulting PCR fragments were cloned into maintainer and inducer plasmids, which were used as templates for gene replacement PCR (see yeast strains and methods, above).

#### In vivo prion assays

For aggregation, inducers were overexpressed by growth of cells in selective medium with 50 μM CuSO_4_ until the culture reached stationary phase; cells were then examined by fluorescent microscopy (Zeiss Axiovert, Zeiss, Oberkochen, Germany; Metamorph imaging software, Universal Imaging Corporation, Downingtown, Pennsylvania, United States). Unless otherwise noted, cultures displaying 10% or more cells with foci were scored as positive. For induction, dilutions of the above cultures were plated onto SD-ade and YEPD media to determine percentage of Ade+. In qualitative assessments, strains were scored as positive if 5% or more of plated cells grew on SD-ade medium after 5 d. In [*NU^+^*] maintenance experiments, strains with indicated maintainers were tested for the ability to support an Ade+ state following New1_1–153_-GFP overexpression. In [*PSI^+^*] maintenance experiments, strains that began as [*PSI^+^*] were tested for Ade+ after plasmid shuffle gene replacement with the indicated maintainer. For decoration, a [*PSI^+^*] [*PIN^+^*] strain was transformed with the indicated inducers, grown in selective medium with 50 μM CuSO_4_, and examined by fluorescence microscopy during midlogarithmic phase. Propagon counts were performed as described in Cox et al. (2003). For the antisuppression assay, indicated strains were transformed with a second, differently marked maintainer plasmid, and color phenotypes were assayed on medium selective for both plasmids.

#### In vitro prion assays

Centrifugation was performed as described in Ness et al. (2002). Immunoblots were visualized with MT130 anti-Sup35p N-M domain serum.For the polymerization of PNM2-1, the PNM2-1 N and M domains were cloned as 7-histidine fusions into pAED4 and expressed and purified as described in DePace et al. (1998). Thioflavin-T binding was conducted as in Chien et al. (2003). The slope of early (0–6 min) dye binding was obtained from seeded polymerization reactions conducted in triplicate. To correct for a difference in dye binding between wild-type and PNM2-1 protein, these values were normalized to the end point (90 min) maximum signal for each protein. Monomer concentrations were 2.5μM.

## Supporting Information

Data S1 DNA Sequences of Constructs (30 KB ZIP).

### Accession Numbers

The GenBank accession numbers for the proteins discussed in this paper are Hsp104p (NP_013074), New1p (NP_015098), Rnq1p (NP_09902), Sup35p (NP_010457), Ure2p (NC_014170), YDR210W (NP_010496), and YBR016W (NP_010319).

## Abbreviations

Ade+: adenine prototrophy
C: carboxy-terminal
GFP: green fluorescent protein
GuHCl: guanidine hydrochloride
M: middle
N: amino-terminal
NYN: asparagine-tyrosine-asparagine
Q/N: glutamine/asparagine
PNM: PSI No More
SD: synthetic defined (yeast medium with dextrose)
YEPD: yeast extract-peptone-dextrose
